# ALG8-CDG: novel patients and review of the literature

**DOI:** 10.1186/s13023-015-0289-7

**Published:** 2015-06-12

**Authors:** Michaela Höck, Karina Wegleiter, Elisabeth Ralser, Ursula Kiechl-Kohlendorfer, Sabine Scholl-Bürgi, Christine Fauth, Elisabeth Steichen, Karin Pichler, Dirk J. Lefeber, Gert Matthjis, Liesbeth Keldermans, Kathrin Maurer, Johannes Zschocke, Daniela Karall

**Affiliations:** Medical University of Innsbruck, Clinic for Pediatrics II, Division of Neonatology, Innsbruck, Austria; Medical University of Innsbruck, Clinic for Pediatrics I, Inherited Metabolic Disorders, Anichstrasse 35, 6020 Innsbruck, Austria; Division of Human Genetics, Department of Medical Genetics, Molecular and Clinical Pharmacology, Medical University of Innsbruck, Innsbruck, Austria; Department of Neurology, Translational Metabolic Laboratory of Genetic, Endocrine and Metabolic Diseases, Radboud University Medical Center, Nijmegen, The Netherlands; Center for Human Genetics of the University of Leuven, Leuven, Belgium; Department of Radiology, Medical University of Innsbruck, Innsbruck, Austria

**Keywords:** Congenital disorders of glycosylation, Isoelectric focusing of transferrin, Hydrops fetalis, Cataract, Coagulopathy, Gastrointestinal symptoms

## Abstract

**Background:**

Since 1980, about 100 types of congenital disorders of glycosylation (CDG) have been reported representing an expanding group of inherited disorders. ALG8-CDG (= CDG-Ih) is one of the less frequently reported types of CDG, maybe due to its severe multi-organ involvement with coagulation disturbances, edema, massive gastrointestinal protein loosing enteropathy, cataracts, and often early death. We report three additional patients, provide an update on two previously reported, and summarize features of ten patients reported in literature.

**Results:**

Of 15 ALG8-CDG patients, three were homozygous and 12 compound heterozygous. There were multiple prenatal abnormalities in 6/12 patients. In 13/15, there were symptoms at birth, 9/15 died within 12 months. Birth weight was appropriate in 11/12, only one was small for gestational age. Prematurity was reported in 7/12. Hydrops fetalis was noticed in 3, edemas in 11/13; gastrointestinal symptoms in 9/14; structural brain pathology, psychomental retardation, seizures, ataxia in 12/13, muscle hypotonia in 13/14. Common dysmorphic signs were: low set ears, macroglossia, hypertelorism, pes equinovarus, campto- and brachydactyly (13/15). In 10/11, there was coagulopathy, in 8/11 elevated transaminases; thrombocytopenia was present in 9/9. Eye involvement was reported in 9/14. CDG typical skin involvement was reported in 8/13.

**Conclusion:**

In ALG8-CDG, isoelectric focusing of transferrin in serum or plasma shows an abnormal sialotransferrin pattern. The diagnosis is confirmed by mutation analysis in *ALG8*; all patients reported so far had point mutations or small deletions. The prognosis is generally poor. Thus, a timely and correct diagnosis is important for counselling.

## Background

Congenital disorders of glycosylation (CDG) involve a rapidly growing group of metabolic diseases that are caused by genetic defects in the synthesis of glycoproteins [1]. The first two patients were diagnosed in 1980 by the Belgian pediatrician Jaak Jaeken [2]. To date, some 100 CDG have been identified [1, 3] (with over 700 patients reported worldwide), and about 50 of these can be identified via isoelectric focusing of transferrin [1, 3–5]. CDG have been divided in four groups: (1) disorders of protein N-glycosylation, (2) disorders of protein O-glycosylation, (3) disorders of lipid and GPI (glycosylphosphatidylinositol) anchor glycosylation, and (4) disorders of multiple glycosylation pathways [5, 6]. Defects in protein N-glycosylation are divided into two subgroups: CDG-I disorders comprise defects in the glycan synthesis (cytosol and endoplasmic reticulum (ER)), and CDG-II disorders defects in glycan processing (Golgi apparatus) [1]. In 2008, Jaeken *et al.* proposed a nomenclature that refers to the gene name under the umbrella name of CDG, *e.g.* PMM2-CDG, ALG8-CDG, *etc.* [7]. CDG affect multiple organ systems, the severity of symptoms is highly variable and it shows a broad clinical spectrum with considerable overlap [1, 3].

ALG8-CDG (OMIM #608104) is due to inactivating mutations in the *ALG8* gene encoding dolichyl-phosphate-glucose 1-mannose 9-N-acetylglucosamine (Dol-P-Glc:Glc(2)Man (9)GlcNAc(2)-PP-Dol-alpha-2) glucosyltransferase (= glucosyltransferase 2) in the ER, an enzyme that attaches the second glucose residue to dolichol-PP-glycans in the ER [8]. ALG8-CDG is a CDG form with dysmorphism (retrognathia, low-set ears, pes equinovarus), muscular hypotonia, hepatomegaly, coagulopathy (thrombocytopenia), edema and ascites (including fetal hydrops), cardiorespiratory problems, and protein-losing enteropathy (watery diarrhea) [8]. Cataracts (ocular findings) have only been reported in ALG8-CDG, ALG12-CDG (CDG-Ig), other subtypes in early LLO (lipid-linked oligosaccharides) synthesis (like SRD5A3-CDG) and some unclassified CDG [9, 10].

Here, we describe three severely affected neonates from two families with ALG8-CDG with congenital cataracts, and provide an update on two previously reported children with congenital cataracts [11], where ALG8-CDG was confirmed recently. In addition, we summarize the clinical and biochemical characteristics of these five patients, further ten published patient reports and present a review of the literature.

### Clinical reports - patients

#### Patient 1

The first child of healthy, non-consanguineous, Caucasian parents, was born in the 34th gestational week after oligohydramnios in pregnancy. 15 min after birth, the boy died of severe respiratory failure due to pulmonary hypoplasia with alveolar dysgenesis. Clinical examination showed low-set ears, macroglossia, bilateral pes equinovarus, hypoplastic thorax, generalized edema, and a pale gray mottled skin. Bilateral cataracts were noted. Autopsy showed renal tubular dysgenesis, cerebral edema and ascites.

#### Patient 2

The second boy, brother of Patient 1, was born at 35 weeks of gestation by Caesarean section because of a pathological cardiotocogram (CTG). Pregnancy was complicated by intrauterine hydrops fetalis with pericardial effusion and ascites as well as oligohydramnios. Birth weight was 2700 g (50th centile), head circumference 34.5 cm (75th centile) and length 48 cm (75th centile). The Apgar scores were 3/7/8 and umbilical cord pH was 7.36. Because of respiratory insufficiency, the boy was mechanically ventilated for four days. Dysmorphism (wide-open fontanel, incomplete bilateral cataract, low-set ears, hypertelorism, macroglossia, retrognathia), generalized edema, a pale mottled skin as well as pronounced muscular hypotonia were present. Laboratory investigation showed thrombocytopenia (lowest value 19,000/μL, decreased prothrombin time (PT)(40 %; nl 74–108) and increased partial thromboplastin time (PTT)(>200 s.; nl 28–49)).

After initial stabilization the patient’s general condition worsened in the third week of life. He developed watery diarrhoea and vomiting, and subsequently acute renal failure with anuria and ascites. His condition deteriorated and at 34 days of life he died due to cardiocirculatory failure.

#### Patient 3

In 1996, our group reported the first, albeit at that time unclassified, ALG8-CDG patient (Table 1) [11]. The girl was the second child of healthy, unrelated, Caucasian parents and died at the age of 30 months. She presented with severe psychomotor disability, microphthalmy, cataract, brachydactyly, cortical atrophy, slight elevation of serum transaminases and blood coagulation abnormalities.

**Table 1:** Findings in 15 patients with ALG8 deficiency

	Skladal [11]		Charlwood [14]	Chantret [8]	Schollen [15]		Eklund [9]	Vesela [16]	Stölting [17]		Sorte [18]	Funke [19] Kouwenberg [20]	Patients 1, 2, 5		
Sex	F	M	M	F	F	M	M	F	M	F	F	M	M	M	F
**Prenatal Data**															
IUGR	/	/	Yes	/	Yes	No	No	No	No	No	Yes	No	No	No	No
Oligohydramnios	/	/	Yes	/	Yes	No	No	Yes	No	No	No	No	Yes	Yes	No
Hydrops fetalis	/	/	ND	/	/	/	/	Yes	No	No	/	No	Yes	Yes	No
**Neonatal data**															
Weeks of gestation	/	36 w (CS)	35 w (CS)	/	/	35 w	37 w	29 w (CS)	At term	At term	39 w (CS)	35 w	34 w (CS)	35 w (CS)	37 w (CS)
Birth weight	/	2,590 g	2,280 g	/	/	2,920 g	3,070 g	1,420 g	3,980 g	3,680 g	2,210 g	2,920 g	2,570 g	2,700 g	3,080 g
**Clinical symptoms**															
Ascites/edemas	/		Yes	Yes	Yes	Yes	Yes	Yes	No	No	Yes	Yes	Yes	Yes	Yes
Onset of first symptoms	From birth		From birth	4 months	From birth	8 weeks	From birth	From birth	From birth	From birth	From birth	From birth	From birth	From birth	From birth
Dysmorphism	Yes		Yes	No	Yes	Yes	Yes	No	Yes	Yes	Yes	Yes	Yes	Yes	Yes
Gastrointestinal symptoms	/		Yes	Yes	No	Yes	Yes	Yes	No	No	Yes	Yes	No	Yes	Yes
Brain involvement	Yes		Yes	No	/	Yes	Yes	Yes	Yes	Yes	Yes	Yes	/	Yes	Yes
Hypotonia	/		Yes	No	Yes	Yes	Yes	Yes	Yes	Yes	Yes	Yes	Yes	Yes	Yes
Skin involvement	/		Yes	No	/	Yes	No	No	Yes	Yes	Yes	Yes	No	No	Yes
Eye symptoms	Cataract		No	Retinopathy	/	No	Cataract	Optic atrophy	No	No	Nystagmus	No	Cataract	Cataract	Cataract
**Laboratory data**															
Thrombocytopenia	/		Yes	/	Yes	Yes	Yes	Yes	/	/	Yes	Yes	/	Yes	Yes
Coagulopathy	Yes		Yes	Yes	Yes	ND	Yes	Yes	/	/	Yes	Yes	/	Yes	No
Hyponatremia	/		Yes	/	Yes	Yes	Yes	/	/	/	/	Yes	Yes	Yes	Yes
Increased transaminases	Yes		Yes	No	/	Yes	Yes	No	/	/	Yes	Yes	/	Yes	No
**Survival**	30 months	4 years	3 months	>3 years	3 days	3 months	16 months	2 months	>6 years	>7 years	8,5 months	3 months	15 min	34 days	39 days
**ALG8 mutations**	/	Homozygous	Comp. het.	Comp. het.	Comp. het.	Comp. het.	Homozygous	Comp. het.	Comp. het.	Comp. het.	Comp. het.	Comp. het.	/	Comp. het.	Comp. het.
Mutation 1 (genomic)		c.139A>C	c.139A>C	c.413delC	c.139A>C	c.673+4A>G	c.139A>C	c.139A>C	c.845C>T	c.845C>T	c.799T>C	c.673+4A>G		c.139A>C	c.139A>C
Mutation 1 (functional)		p.T47P	p.T47P	p.T138Kfs*19	p.T47P	Splice mutation	p.T47P	p.T47P	p.A282V	p.A282V	p.S267P	Splice mutation		p.T47P	p.T47P
Mutation 2 (genomic)		c.139A>C	c.96-2A>G	c.396insA	c.96-2A>G	c.824G>A	c.139A>C	c.1090C>T	c.1436delC	c.1436delC	c.808T>C	c.824G>A		c.1090C>T	c.1219_1220delCT
Mutation 2 (functional)		p.T47P	Splice mutation	p.V133Sfs*3	Splice mutation	p.G275D	p.T47P	p.R364*	p.P479Lfs*6	p.P479Lfs*6	p.F270L	p.G275D		p.A364*	p.L407Dfs*23

*CS* Cesarean section; *ND* not described or no information available; dysmorphism such as low-set ears, macroglossia, pes equinovarus, campto- and brachydactyly; gastrointestinal symptoms include diarrhea, vomiting, protein-losing enteropathy; CNS defects include structural brain abnormalities, mental or psychomotor retardation, seizures; hypotonia presenting as floppy infant; skin involvement is abnormal fat distribution, wrinkly skin, cutis laxa, inverted nipples; electrolyte disturbances refers to hyponatremia; exitus, death, when written as > years it means age at last follow-up, no further information on outcome, comp. het., compound heterozygous

#### Patient 4

**Fig. 1:**
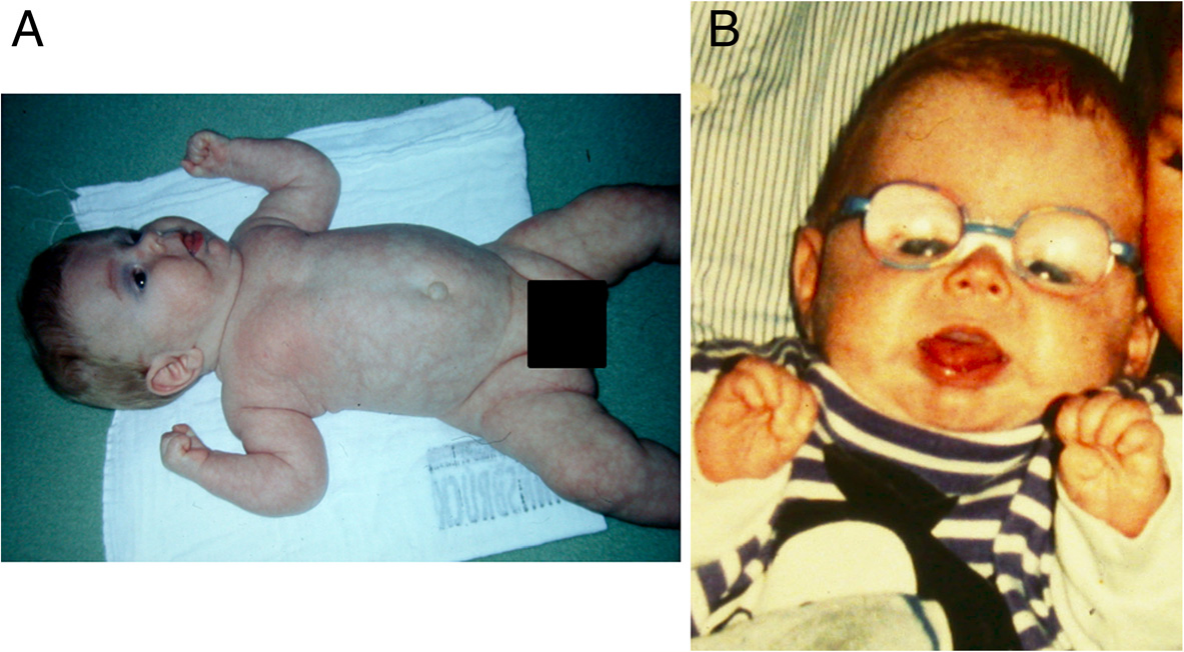
**a**,**b** Patient 4 at the age of 10 months - floppy infant, with multiple dysmorphic features such as a prominent forehead, wide fontanelle, macroglossia, dysplastic ears, high palate, inverted nipples, short fingers and toes and a pale mottled skin, “fat pads” on arms and thighs [11]

Six years later, a brother of Patient 3 (Fig. 1a,b) [11] was born after an uneventful pregnancy by Caesaran section in the 36th gestational week because of breech presentation. Birth weight was 2590 g, length 48 cm and head circumference 33 cm (all 25th centile). The boy presented as a floppy infant, with bilateral congenital cataracts and multiple dysmorphic features such as a prominent forehead, a wide fontanelle, macroglossia, dysplastic ears, a high-arched palate, inverted nipples, peculiar fat pads of the buttocks, and short fingers and toes. Cyanotic apnoeas were repeatedly present from the 4th day of life. Laboratory tests showed slight elevation of transaminases and transient hypoalbuminaemia, as well as pathological coagulation (with prolonged PTT (partial thromboplastin time), low antithrombin III, and several reduced coagulation factors). He suffered from vitreous bleeding during cataract operation and died at the age of four years from cardiac dysrhythmia.

#### Patient 5

**Fig. 2:**
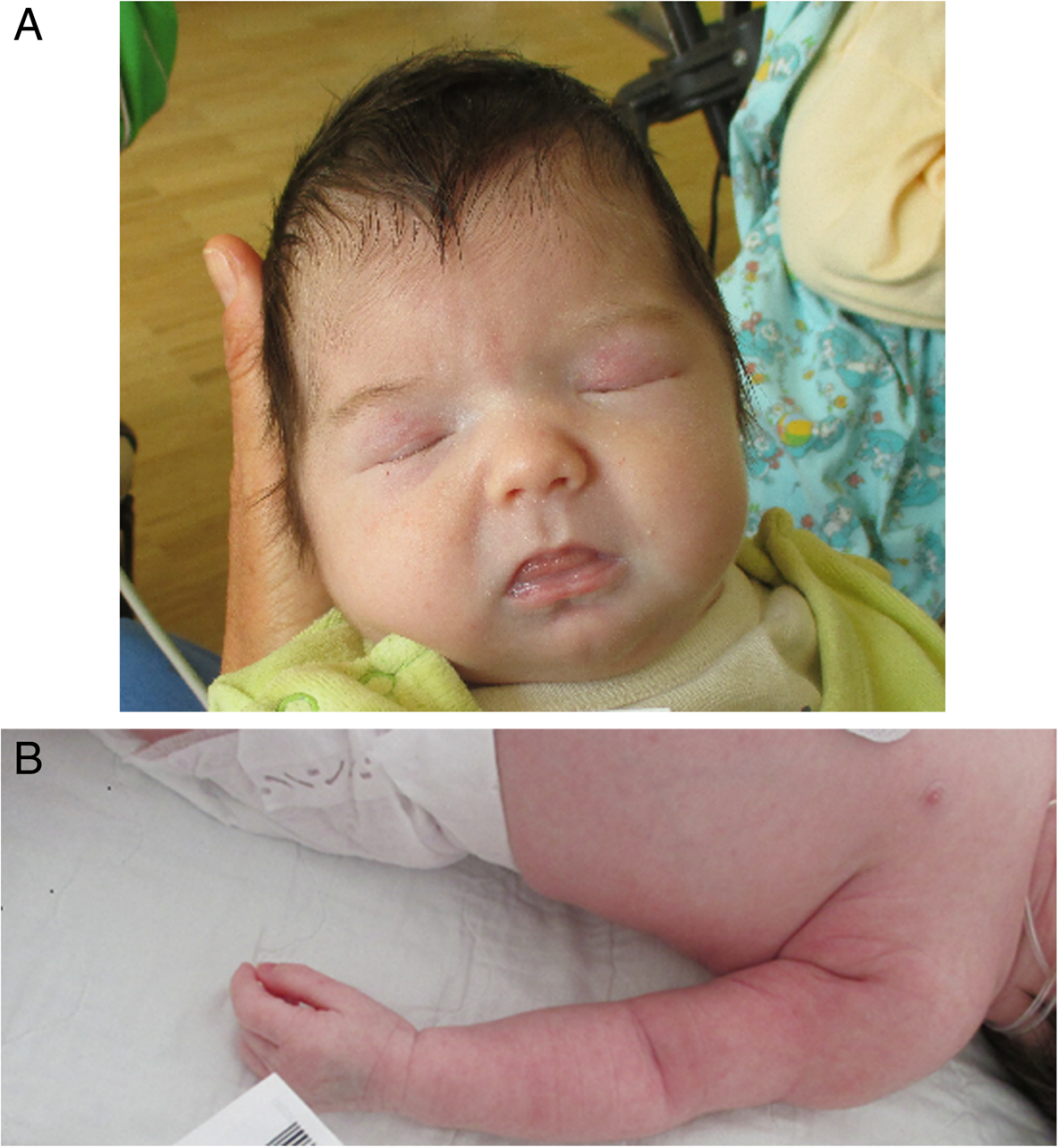
**a**,**b** Patient 5 at the age of 20 days - floppy infant, with intermittent dystonic posturing, and multiple dysmorphic features such as an abnormal fat distribution on arms and thighs, macroglossia, low-set ears, additionally, cataracts and persisting primary vitreous artery were present

**Fig. 3:**
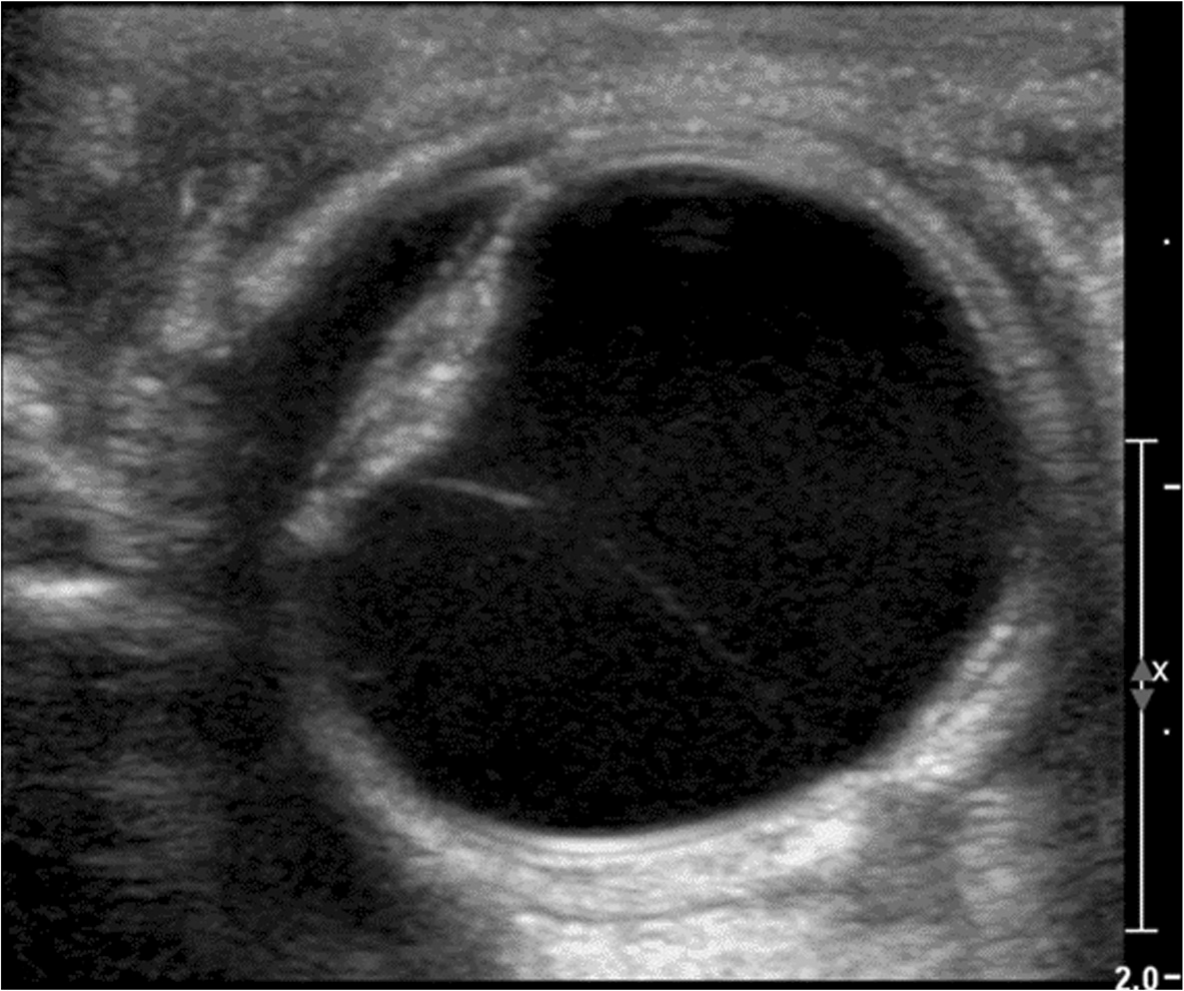
Ocular sonography of Patient 5 at the age of 10 days - Persistent hyperplastic primary vitreous artery. A thin y-shaped membrane extends from the optic disc to the posterior aspect of the opacified lens representing a remnant of the hyaloid vasculary system

This girl (Fig. 2a), sister of a healthy four year old girl, was born in the 37th gestational week after an uneventful pregnancy. Labour was induced because of a pathological CTG. Birth weight was 3080 g (50th centile), length 47 cm (10th centile) and head circumference 33 cm (25th centile), the Apgar scores were 8/9/10 and arterial umbilical cord pH was 7.36. She was hospitalized due to fluctuations of saturation, tachypnea and increased inflammation parameters as well as thrombocytopenia (lowest platelet count was 49 G/l; normal range: 223–510 G/l), and was treated with antibiotics. During the first days, she vomited repeatedly. She showed mild microcephaly, facial dysmorphism (bilateral cataract, low-set ears, relative macroglossia), fat pads on the arms (Fig. 2b) and thighs, hypotonia with dystonic posturing, hepatosplenomegaly, and persisting primary vitreous artery (Fig. 3). Routine laboratory parameters (including coagulation parameters and thyroid function tests) were normal. Over the next weeks, the girl deteriorated progressively with recurrent episodes of apneas often combined with bradycardia. Electroencephalogram (EEG) showed a pathological brain wave pattern with decreased brain activity. On day 38 of life, a pale skin and apneas were noted, with ventricular tachycardia. The girl died from cardiovascular arrest.

## Methods

Serum transferrin isoelectrofocusing was performed as reported [12]. Mutation analysis of the *PMM2* and *ALG8* genes was performed according to standard methods [1, 13].

In CDG, enzyme analyses are not available for all types of defects in the pathway. When enzymatic analysis lacks, lipid-linked oligosaccharides (LLO) in fibroblasts can show characteristic profiles [13], narrowing down the diagnosis of CDG. For confirmation of the diagnosis, molecular genetic testing is necessary. Standard Sanger sequencing was performed in all five patients. In the future, with a better availability, exome or whole genome sequencing methods might be helpful as well.

## Results

In all our 5 patients, the plasma isoelectric focusing of transferrin showed a type 1 pattern (CDG-I) and confirmed the clinical suspicion of an underlying CDG.

In Patient 4, sialotransferrins showed a type 1 pattern (CDG-Ix). However, PMM activity in fibroblasts was normal. Through enrollment in the Euroglycanet network, ALG8-CDG was diagnosed in 2011 (homozygous c.139A>C p.Thr47Pro). The parents are heterozygous.

In Patient 2 with prenatal hydrops, edema and cataract, the serum transferrin type 1 pattern prompted us to investigate *ALG8* gene as a candidate. A founder mutation was suspected as the family originated from the same Tyrolean region where we previously had diagnosed another pair of siblings (Patient 3 and 4) with type 1 pattern and cataract [11]. Two heterozygous mutations in the *ALG8* gene were found in DNA extracted from fibroblasts of Patient 2 (c.139A>C in exon 2, p.Thr47Pro, and c.1090C>T in exon 10, p.Arg364Ter), confirming the diagnosis of ALG8-CDG.

The clinical similarity and plasma transferring type I pattern prompted analysis of *ALG8* gene in Patient 5. This showed compound heterozygosity for the known *ALG8* mutation in exon 2, c.139A>C, p.Thr47Pro and a newly described one in exon 11, c.1219_1220delCT, p.Leu407Aspfs*23.

Both the c.139A>C and c.1090C>T mutations are known disease causing mutations described previously, c.1219_1220delCT is a frame shift mutation with subsequent premature stop codon formation.

Of the 15 patients summarized here (3 newly described, 12 reported in literature [8, 9, 14–20] – including 2 published by our group [11]), three are homozygous (including one pair of siblings) and 12 compound heterozygous (including three pairs of siblings). Of the 26 allels detected, 9 (35 %) are c.139A>C (Table 1). If we add the analyses of the deceased siblings (Patient 1 and Patient 3), there would be 30 alleles in total, and 12 (40 %) c.139A>C.

In summary, Table 1 shows features of 15 ALG8-CDG patients in total. There are multiple prenatal abnormalities in 6/12 patients: 3 had intrauterine growth retardation, 5 oligohydramnios and 3 hydrops fetalis. In 13/15 patients symptoms were recorded at birth and 9/15 died within the first year of life, showing the severity of ALG8 deficiency. Birth weight was appropriate for gestational age in 11/12 patients and only one patient was small for gestational age (birth weight <10th centile). Prematurity (<37 weeks) was reported in 7/12 patients. Hydrops fetalis was noticed only in three patients, whereas edema was present in 11/13. Common dysmorphic signs were: low set ears, macroglossia, hypertelorism, pes equinovarus, campto- and brachydactyly (13/15). Gastrointestinal symptoms included diarrhea, vomiting, feeding problems with failure to thrive, protein-losing enteropathy (9/14 patients). Brain involvement included psychomotor disability, seizures, ataxia, and structural abnormalities (dilatation of ventricles, corpus callosum hypoplasia, leukoencephalopathy/leukodystrophy, cortical/cerebral atrophy) in 12/13 patients. Hypotonia/‘floppy infant’ was present in 13/14 patients. Thrombocytopenia was present in 9/9 patients. Most patients had liver pathology: elevated serum transaminases in 8/11 and abnormal clotting factors in 8/11. Eye involvement (especially cataracts) was reported in 9/14 patients and skin involvement like fat pads, wrinkly skin, cutis laxa and inverted nipples was reported in 8/13 patients.

## Discussion

ALG8-CDG is one of the less frequently reported CDG syndromes. It affects an early step of protein glycosylation and in general has a poor prognosis. It was first reported by Chantret *et al.* in 2003 [8]. There are twelve patients described to date (Skladal *et al.* [11] (n = 2), Charlwood *et al.* [14] (n = 1), Chantret *et al.* [8] (n = 1), Schollen *et al.* [15] (n = 2), Eklund *et al.* [9] (n = 1), Vesela *et al.* [16] (n = 1), Stölting *et al.* [17] (n = 2), Sorte *et al.* [18] (n = 1), and one patient reported by both Funke *et al.* [19] and Kouwenberg *et al.* [20] (Table 1). In the majority of patients (13/15), the disease had a neonatal onset and rapid progression. In 9/15 patients, the outcome was fatal within the first year of life (Table 1). So far, no adult patients (>18 years of age) affected with this subtype have been reported [21].

We reviewed the most common clinical, laboratory and genetic findings in the twelve previously reported patients diagnosed with ALG8-CDG (Table 1), add here the data of three additional patients with ALG8-CDG (Patient 1,2 and 5), and provide an update on two previously reported patients (Patient 3 and 4). For the two patients reported by Charlwood *et al.* [14] and the two reported by Skladal *et al.* (Patient 3 and 4) [11], the diagnosis of ALG8-CDG was provided years later.

In the series of 15 patients summarized in this paper, there are four pairs of siblings (Schollen [15] plus the patient reported by Charlwood et al [14], Stölting [17], Patient 1/2, and Patient 3/4 in this paper) (Table 1), reflecting an ascertainment bias for firstborns deceased without diagnosis being established before a second affected child was born.

Hydrops fetalis has also been described in PMM2-CDG and ALG1-CDG [1]. In the patients described here, it was present in the two brothers (Patient 1 and 2) and in 1/12 additional patient reported in the literature (Table 1). Hyponatremia reported in eight patients is probably secondary to ascites/edema (11 patients). Unlike other CDG types, cataracts (ocular findings) have only been reported in ALG8-CDG, ALG12-CDG, other subtypes in early LLO synthesis (like SRD5A3-CDG) and some unclassified CDG [9, 10].

CDG are a clinically and genetically heterogeneous group of disorders. Patients with ALG8-CDG show very similar clinical features with neonatal onset and fatal outcome within the first year of life. We believe that this CDG disorder might be underdiagnosed. Early discriminative symptoms in ALG8-CDG patients are multisystem involvement with dysmorphism, neurological symptoms and cataracts in about 50 % of cases. Therefore, we suggest screening for CDG sialotransferrins in serum or plasma in all infants with early multi-organ involvement (such as cataracts, non-immune hydrops, coagulopathy). Confirmation of diagnosis requires molecular genetic testing.

The diagnosis of CDG is frequently delayed due to the highly variable phenotype, with some cases showing single organ involvement and others mimicking syndromes, like skeletal dysplasia, cutis laxa syndrome, or congenital muscle dystrophy.

In MPI-CDG (=CDG-Ib) (phosphomannose isomerase deficiency), the protein-losing enteropathy and hypoglycaemia can be treated with an oral uptake of mannose [22], in SLC35C1-CDG (=CDG-IIc), the leukocyte adhesion deficiency can be treated with oral fucose [23], and in PGM1 (Phosphoglucomutase 1 deficiency), treatment with galactose leads to improved indexes of glycosylation [24]. For ALG8-CDG, there is currently no curative treatment available. However, establishing a molecular diagnosis is important for family counselling and prenatal diagnosis [25].

### Informed consent

All procedures were in accordance with ethical standards of the Helsinki Declaration. Written informed consent for the case reports and images was obtained from all parents of the five patients reported.

## Abbreviations

ALG8-CDG: Alpha-1,3-Glucosyltransferase CDG
CDG: Congenital disorder of glycosylation
IUGR: Intrauterine growth restriction
AGA/SGA: Appropriate for gestational age/small for gestational age
